# G2O-Pose: Real-Time Monocular 3D Human Pose Estimation Based on General Graph Optimization

**DOI:** 10.3390/s22218335

**Published:** 2022-10-30

**Authors:** Haixun Sun, Yanyan Zhang, Yijie Zheng, Jianxin Luo, Zhisong Pan

**Affiliations:** College of Command and Control Engineering, Army Engineering University of PLA, Nanjing 210007, China

**Keywords:** monocular 3D human pose, general graph optimization, multiple constraints, real-time

## Abstract

Monocular 3D human pose estimation is used to calculate a 3D human pose from monocular images or videos. It still faces some challenges due to the lack of depth information. Traditional methods have tried to disambiguate it by building a pose dictionary or using temporal information, but these methods are too slow for real-time application. In this paper, we propose a real-time method named G2O-pose, which has a high running speed without affecting the accuracy so much. In our work, we regard the 3D human pose as a graph, and solve the problem by general graph optimization (G2O) under multiple constraints. The constraints are implemented by algorithms including 3D bone proportion recovery, human orientation classification and reverse joint correction and suppression. When the depth of the human body does not change much, our method outperforms the previous non-deep learning methods in terms of running speed, with only a slight decrease in accuracy.

## 1. Introduction

Estimating a 3D human pose from a monocular view is a research hotspot in the field of computer vision [1]. It has been widely used in human–computer interaction [2], action recognition [3], pedestrian reidentification [4], etc. Monocular 3D human pose estimation has attracted extensive attention due to its simple equipment and flexible application. Mainstream 3D human estimation methods can be divided into traditional methods and deep learning methods. Deep learning methods have high accuracy and speed, but have the following disadvantages: dataset dependency and high computational power requirement. Although traditional methods are not as accurate as deep learning methods, the efforts of many researchers have greatly improved their accuracy (MPJPE: 74 mm [5]). The advantages of traditional methods are: they do not require training, nor do they rely too much on datasets. The main problem of the current traditional methods is that their speed is far from real time, which limits their wide application.

In recent years, 2D human pose estimation technology has been gradually maturing. Both single-player pose estimation [6,7] and multiplayer pose estimation (OpenPose [8], CPN [9]) have shown good performance. Therefore, 3D human pose estimation from a 2D pose has become the mainstream method. Along this direction, in this paper, we propose a real-time 3D human pose estimation method called G2O-pose, based on graph optimization framework. A variety of constraints are imposed on the 3D pose in every single frame to avoid ambiguity. Under the circumstance of little change of human body depth (less than 0.3 m in our experiments), our method has similar accuracy and greatly improved speed compared with previous traditional methods. On our laptop with an Intel i7 1.8 G CPU, 8 GB RAM and a GeForce MX250 GPU, the FPS (frames per second) of the algorithm from 2D keypoints to 3D pose reasoning reaches 32 (for single player). As the interval sampling, the processing speed of the original 2D poses sequence reaches more than 100 fps. The main contributions of this paper are as follows:

1. Bone proportion recovery algorithm based on multiple 2D poses. No matter how the human body moves, the proportions and lengths of the human bone segments remain unchanged. Aiming at this feature, this paper studies the algorithm of recovering the proportions of the 3D bone segments from multiframe 2D poses. The average proportion error of the bones is 0.012 (calculated by the ground truth 2D keypoints, and the spine segment is used as the proportion 1).
2. Three-dimensional human orientation classification algorithm based on 2D joint features. Aiming at the problem of the depth ambiguity of two shoulders (or hips) keypoints, the 3D orientation of human body is estimated by 2D pose features. The accuracy of the classification reaches 95.4% (ground truth 2D key points).
3. Reverse joint correction algorithm based on heuristic search. The front and back of joint rotation are difficult to be distinguished in 2D images. Therefore, the detection, correction and suppression algorithm of the reverse joints based on rotation angles are studied, and the average accuracy of the corrections is more than 80%.
4. Three-dimensional human pose estimation method based on graph optimization under multiple constraints. The 3D keypoints of human body are modeled as a graph. Under the constraints of algorithm 1–3, the 3D pose of the next frame is solved in turn with the previous pose as initial solution. The accuracy of our method is comparable to that of the previous traditional methods while the speed is greatly improved, when the depth of the human body does not change so much.

This rest of this paper is arranged as follows: Section 2 introduces related works; Section 3 is our method; Section 4 is the experimental results. Section 5 is discussion; In Section 6, we made a summary.

## 2. Related Works

Three-dimensional human pose estimation, especially from monocular view, is still a complex problem in computer vision. Because human motion is highly nonlinear and nonrigid, the inference from 2D to 3D is challenging. 3D human pose estimation can be divided into traditional methods and deep learning methods.

### 2.1. Traditional Methods

The traditional 3D human pose estimation methods can be further divided into three categories: generative approaches (model-based), discriminative approaches (non-model-based), and hybrid approaches.

#### 2.1.1. Generative Approaches

Generative approaches utilize pregenerated human models, also known as model-based methods. They are usually divided into two stages. The first stage is modeling, that is, constructing likelihood function. By considering all the aspects of the problem such as the image descriptors, the structure of the human body model and the camera model, the likelihood function is constructed according to the Bayes rule. The second stage is reconstruction, i.e., solving the 3D human pose according to the likelihood function [10]. The most typical model is the pictorial structure model (PSM [11]). It regards the human body as a collection of body segments connected by joints, and calculates the 3D pose of by solving the poses of each part. Ref. [12] proposed an improved model of PSM: the deformable structure, which regards the human body as a nonrigid body whose shape can be changed and can model the human body in more detail. This kind of method depends heavily on the accuracy of the initial 3D pose, and it is easy to lose track in the following process.

The advantage of generative approaches is that the human body is generated with high accuracy with appearance and deformation, such as clothing and accessories. However, the disadvantages are large amount of computation, high computational complexity and slow speed.

#### 2.1.2. Discriminative Approaches

Discriminative approaches do not need the human body model established in advance, also called as non-model-based approaches.

They calculate 3D poses by establishing the mapping relationship between 2D and 3D. They can be further divided into learning-based methods and example-based methods [10]. Learning-based methods establish the mapping relationship from image to 3D pose through learning, and usually have good generalization ability [13,14]. Example-based methods are to construct 3D human pose libraries, and final calculated 3D poses are obtained by combining and interpolating the instances in the library. Because the space of the established instance library is usually small, this kind of method is fast and robust. The authors of [15] propose a method to reconstruct 3D human poses under a chaotic background, and use a support vector machine (SVM) and linear regression to obtain 3D poses. The author of [16] proposes a method based on massive instances, which searches the instance database through K-D tree to reconstruct reasonable 3D poses. The authors of [17] propose a sparse probability regression method independent of activity, which uses a sparse Gaussian process to improve the accuracy of multimodal output. The authors of [18] propose a classification-based method, which regards the whole human body as a combination of 3D poses, and obtains poses by classifying the input gradient histogram feature vectors. In ref. [19], a sparse representation method is proposed, which represents a large number of poses with a small number of basic poses and reduces the computational cost. The authors of [20] propose a method based on factorization to recover the shape of a nonrigid body (NRSFM) from a monocular video sequence. The authors of [21] also propose a convex optimization algorithm based on sparse representation, and adopt the learning method to construct a 3D human pose dictionary. This method is used to initialize the pose in following studies. In ref. [5], two optimization algorithms are proposed, respectively, for cases whether the 2D keypoints are known. When the 2D keypoints are given, optimization methods such as cyclic coordinate descent are used to solve the problem directly. When the 2D keypoints are unknown, the expectation maximization algorithm (EM), which regards them as hidden variables, is adopted. MPJPE reaches 74 mm tested on the Human3.6M dataset [22].

Discriminative methods use simple representations for human body, so their advantages lie in faster operating speed and lower computation cost. Compared with generative methods, the performance is less dependent on feature sets or inference methods [23].

#### 2.1.3. Hybrid Approaches

There are approaches combining generative approaches and discriminative approaches. They can reconstruct a 3D human pose more accurately. The likelihood function is constructed by using the generative method, and then the mapping function of the discriminative method is verified by the likelihood function. The authors of [24] propose a unified framework for 3D surface and articulated pose reconstruction.

### 2.2. Deep Learning-Based Methods

Early methods mainly solve 3D human poses by optimization methods under constrained conditions. In recent years, 3D human pose estimation based on deep learning has made great progress due to the great development of neural networks and large datasets (e.g., HumanEva [25], Human3.6m [22]). Most mainstream deep neural networks are implemented by designing encoders and decoders (e.g., [26,27,28,29,30]). Encoders mainly realize high-order feature extraction, while decoders mainly realize 3D keypoints or 3D human mesh generation. Since deep learning methods mostly follow this framework, how to improve the estimation accuracy is translated into designing more reasonable network structures, such as CNN, RNN, GCN, Transformer, etc. [1].

Deep learning methods have achieved better performance in accuracy and speed compared with traditional ones. However, deep learning methods also have drawbacks, such as the dependence on datasets and high computational cost. Most existing deep learning methods are difficult to be deployed on resource-constrained edge devices. Traditional ones are far from real-time performance

In this paper, we propose a lightweight real-time 3D pose estimation method that can run quickly on consumer computers with comparable accuracy of traditional methods.

## 3. Algorithm

In this paper, we implement 3D human body pose estimation under constraints of the 2D keypoints and other constraints. Two-dimensional keypoints can be extracted by CPN [9] or ones supplied with the dataset can be used. Our method can be classified into the discriminative approaches due to just using simple keypoints to represent 3D poses.

**Figure 1:**
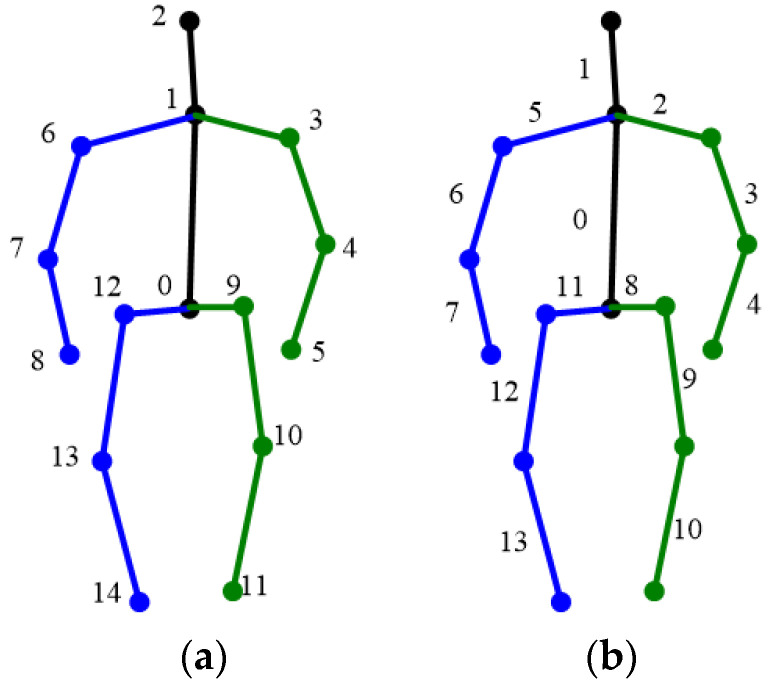
Three-dimensional pose model in this paper. (**a**) Three-dimensional keypoints; (**b**) bone segments. Keypoints: 0 Waist, 1 Neck, 2 Head, 3 Left Shoulder, 4 Left Elbow, 5 Left Wrist, 6 Right Shoulder, 7 Right Elbow, 8 Right Wrist, 9 Left Hip, 10 Left knee, 11 Left Ankle, 12 Right Hip, 13 Right Knee, 14 Right Ankle.

The constraints used include 3D skeleton length, 3D human body orientation and the rotation angle of the joints. In the following paragraphs, the word with first letter capitalized represents a 3D keypoint (e.g., Waist, Neck). There are 15 keypoints and 14 bone segments in model adopted in this paper, shown in Figure 1:

The dataset used in this paper is Human3.6m [22]. It includes the motion videos of 5 female and 6 male subjects from 4 perspectives and the corresponding ground truth 3D keypoint coordinates.

The error evaluation metric is the mean per joint position error (MPJPE), which is the most widely used metric. After the root point (usually the Waist keypoint) of calculated pose is aligned with that of the ground truth, the mean Euclidean distances between other keypoints are measured.

Our algorithm is as shown in Algorithm 1.

**Algorithm 1:** Algorithm G2O-Pose**Input.** 2D poses, camera parameters. **Output.** 3D poses. **Step 1.** Preprocess: calculate 3D bone segment proportions. Suppose the depth of the body (randomly) and calculate the lengths of bone segments. **Step 2.** Initialize the G2O optimizer, and the initial 3D pose. While (the depth change of the body is less than T), repeat Step 3–Step 7. **Step 3.** Calculate 3D body orientation. **Step 4.** Correct reverse joints of legs. **Step 5.** Solve 3D coordinates of the legs by optimization with the previous 3D pose as initial solution. **Step 6.** Correct reverse joints of the spine and arms. **Step 7.** Solve 3D coordinates of the upper part of body by optimization.

In Step 1, the 3D bone proportions are recovered by using data of multiple frames and multiple actions. Three-dimensional skeleton lengths do not refer to the real lengths of bones, due to the real depth of the human body being unknown. Instead, the lengths are calculated with the supposed depth (randomly set) of the body, so that the calculated pose is proportional to the real one. Finally, all bones are scaled to the true size when MPJPE is calculated.

For the initial frame, the “3D pose of the previous frame” is approximated by the back projection of 2D coordinates. Although the 3D points are all located on the same plane at this time, the algorithm tends to converge with human motion and subsequent optimization. T is the artificially set threshold to ensure that the depth change of the human pose in the camera coordinate system is not so large, which is set as 0.3 m in the experiment.

In the next part of this section, we propose 3 algorithms for 3D pose optimization. That is, the 3D bone proportion recovery algorithm, human orientation classification algorithm and reverse joint correction and suppression algorithm. The last subsection is the construction and solution of the graph optimization model based on the above constraints.

### 3.1. Three-Dimensional Bone Proportion Recovery and Length Calculation Algorithm Based on Multiple 2D Poses

In 3D human pose estimation, the proportion of each human bone segment is invariant. Thus, we try to recover 3D bone proportions and lengths to impose constraints on the optimization algorithm.

#### 3.1.1. Three-Dimensional Bone Proportion Recovery

The author of [31] proposed a skeleton proportion reconstruction algorithm based on graph structure path retrieval (GPR), but it depends on the artificial setting of the initial proportion and it is not robust. In this paper, an adaptive bone proportion recovery algorithm based on multiple video frames is proposed. The initial proportions do not need to be set manually in advance.

From single view, some bones look shorter because they are not fully extended. Therefore, it is not conducive to obtain accurate proportions from single frame. Based on some prior knowledge, this paper estimates the 3D skeleton proportions by estimating the 2D skeleton proportions of multiframe images, specifically as follows:

(1) Selecting video frames. It is found that most of the daily movements are upright, and the spine is usually approximately vertical, which can be used as a reference. Therefore, video data with more upright and multiangle posture were selected to estimate different bone segments.

(2) Calculating the proportions from a sequence of frames in single view. The proportions are the ratios of the length of each bone segment to that of the spine. Then, the maximum ratio is taken as the proportion of the bone segment in the current view:

(1) $$s_{i}^{v\;}=\underset{f}{max\;}\frac{l_{i}}{l_{0}}\;\;\;$$

where v denotes the current view, i denotes serial number of the bone, f is the frame serial number, l is the 2D length of the bone segment and $l_{0}$ represents the 2D length of the spine.

(3) The proportion obtained from multiperspectives is averaged, i.e.,

(2) $$s_{i}^{a\;}=\frac{1}{4}\sum_{v=0}^{3}s_{i}^{v\;}\;\;$$

where a indicates the current action number, every action contains data from 4 views.

(4) The proportion obtained from multiactions is then averaged, namely:

(3) $$s_{i}^{\;}=\frac{1}{n}\sum_{a=0}^{n-1}s_{i}^{a\;}\;\;$$

where n represents total action number taken from the dataset and $s_{i}$ is the average ratio of the *i*th bone.

(5) The outliers that largely deviate from the average proportion are eliminated. Let $s_{i}$ and $s_{i}^{a}$ form vectors ***s*** = [$s_{0},s_{1},\ldots s_{13}$], $s^{a}$= [$s_{0}^{a\;},\;s_{1}^{a}$, *…*, $s_{13}^{a}$]; if the distance between $s^{a}$ and ***s*** is larger than the threshold, $s^{a}$ will be eliminated:

(4) $$d\left(s,s_{i}^{a}\right)=\sum_{i}\|s-s_{i}^{a}\|>\alpha$$

where ‖ ‖ means the Euclidean distance, α is the threshold. After eliminating outliers, the final proportion is obtained by reaverage.

#### 3.1.2. Recovery of 3D Bone Length with Preset Depth

In this paper, the length constraint of the 3D skeleton is adopted. The length refers to the relative length, and it is obtained under the condition of setting the depth (randomly) of Waist point. The details are as follows:

(1) Calculate the length of spine from a single frame

The length of the spine is the 3D distance between Neck point and Waist point. However, because the 2D keypoints may have noises, the 3D distance of Shoulder and Hip is added to take the average.

In a certain frame, the homogeneous 2D coordinates are: Left Shoulder $R_{3}$, Right Shoulder $R_{6}$, Left Hip $R_{9}$, Right Hip $R_{12}$, Neck $R_{1}$, Waist $R_{0}$, then the 2D spine length of the current pose is calculated as:

(5) $$L_{0}=\frac{dK^{-1}\left[R_{0}-R_{1}+0.5*\left(R_{3}+R_{6}-R_{9}-R_{12}\right)\right]}{2}$$

where *K* represents the camera intrinsic matrix, *d* is the initial depth of Waist joint; that is, the length of the spine is approximately the average of two distances: one is between Neck and Waist and the other is between Shoulder and Hip.

(2) Calculate the average spine length by multiple frames

After calculating by single-frame method, the average spine length of multiple frames was calculated by average.

(3) Calculate lengths of other bone segments according to the obtained bone proportions, i.e.,
  (6) $$L_{j}=s_{j}*L_{0}$$

### 3.2. Three-Dimensional Human Orientation Classification Algorithm Based on Weighted 2D Joint Features

The depth ambiguity of Shoulder (Hip) point is reflected in the uncertainty of human orientation. That is, the two Shoulders (or Hips) may have different relative depths with the same 2D projections.

**Figure 2:**
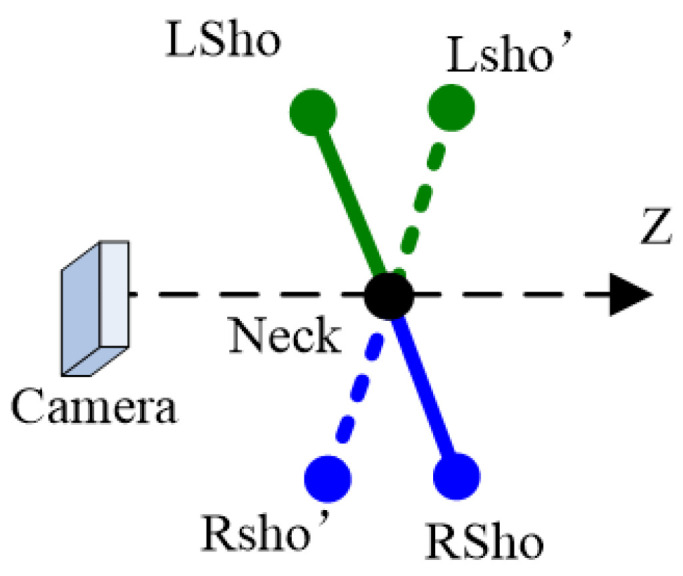
The ambiguity of Shoulders with the same 2D projection.

A top view of the connection between the two Shoulders is shown in Figure 2 (blue for the right limbs, green for the left limbs). The two cases (solid and dashed) will have similar projections. If the direction the camera is pointing is defined as forward, then the body is not sure to go the left or right.

In the meantime, 3D body orientation is closely related to the features of 2D poses. If the body’s face is considered to be forward, it can be inferred that in most cases, Head is in front of Neck, arms are bent forward, and legs are bent backward. In this paper, a 3D human orientation classification algorithm based on weighted 2D joint features is proposed.

The 2D vectors in this subsection are defined in Table 1; all vectors are unitized and have length 1.

Then, we calculate vectors representing bending directions of arms and legs:

(7) $$L_{6,7}=-L_{6}+L_{7}$$

(8) $$L_{3,4}=-L_{3}+L_{4}$$

(9) $$L_{12,13}=-L_{12}+L_{13}$$

(10) $$L_{9,10}=-L_{9}+L_{10}$$

**Figure 3:**
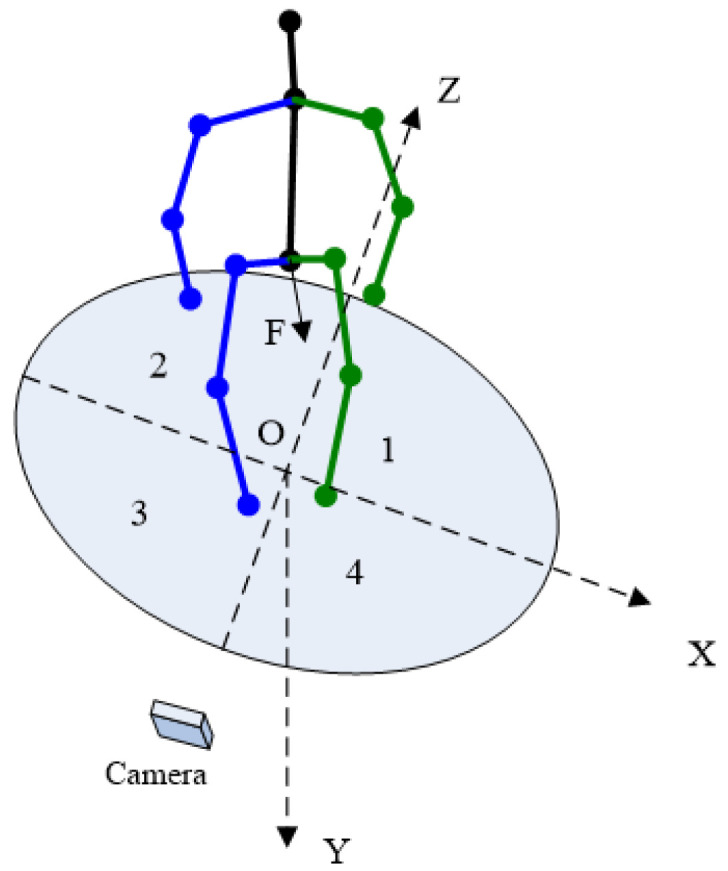
The orientation of the body in coordinate system. The number 1-4 denote the four quadrants of the XOZ plane.

If we establish 3D rectangular coordinate system O-XYZ at where the human body stands, as shown in Figure 3, F is the vector of the body orientation. Since it is already possible to distinguish left or right side of the human body from 2D points, it is easy to judge F vector is pointing in the negative or positive direction of Z axis. The algorithm is mainly concerned with whether F points in the positive or negative direction of the X-axis.

In this paper, a weighted strategy based on 2D vectors is designed to determine the direction of F. Usually $L_{1}$, $L_{6,7}$, $L_{3,4}\;$ are in the same direction as F, while $L_{12,13},L_{9,10}\;$ are in the opposite direction. Thus, we can define:

(11) $$r=w_{1}L_{1x}+w_{2}L_{6,7\left(x\right)}+w_{3}L_{3,4\left(x\right)}-w_{4}L_{12,13\left(x\right)}-w_{5}L_{9,10\left(x\right)}$$

where $w_{1}$, $w_{2},\;\;w_{3},\;\;w_{4}$, $w_{5}$ is the weight coefficient. When r is larger than 0, F vector is considered to point to the positive of X-axis and vice versa.

### 3.3. Reverse Joint Correction Algorithm Based on Heuristic Search

Since there may be multiple 3D poses corresponding to the same 2D pose, the efficiency of the algorithm will be reduced if all poses are evaluated and screened exhaustively. In this subsection, a heuristic search method is adopted. First, we randomly generate a possible 3D pose, and then determine whether it contains reverse joints. If so, the dual keypoints of the 3D keypoints in the depth direction will be judged successively, until the correct one is found.

#### 3.3.1. Reverse Joint Correction Algorithm for Legs

The reverse joint correction algorithm for legs is based on the following two rules:

Firstly, the thighs are mostly forward and the backward rotation does not exceed a certain threshold. If exceeding, they will be corrected to be in front of the body. Taking the left leg as an example, let the unitized 3D thigh vector be $V_{9}$ from Left Hip to Left Knee, and the unitized hip vector $V_{12,9}$ from Right Hip to Left Hip; $V_{0}$ is a unit vector from Neck to Waist, and then a vector pointing to the front of the human body can be obtained:

(12) $$V_{front}=V_{0}\times V_{9,12}$$

where × means cross product. If the projection of $V_{9}$ in the opposite direction of $V_{front}\;$ is greater than the threshold, it will be corrected to the front of the body.

Secondly, when standing, the legs bend in the opposite direction as the front of the body. If the unitized leg bend vector is defined as $V_{c}$, the leg joint should be corrected when the angle between $V_{c}$ and $V_{front}$ is acute.

**Figure 4:**
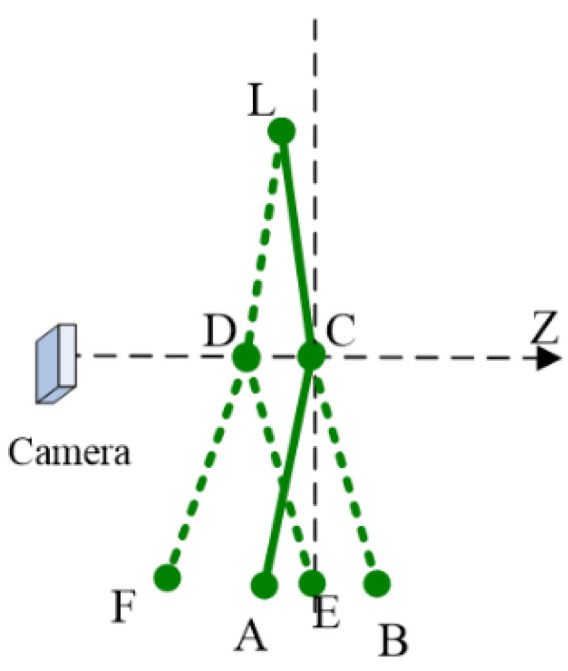
Heuristic search for reverse joint correction. C and D are dual points, i.e., the 3D distance from L is the same to D or to C; in addition, C and D can project to the same position on 2D camera plane.

The correction of a joint is to find the 3D point with the same 2D projection in the depth direction. Still taking the left leg as an example, as shown in Figure 4, If the current gesture of L-C-A contains reverse joint, L-C-B, L-D-E, L-D-F will be successively judged to find the correct one.

When we know the 3D coordinates of A and C, the coordinates of B can be obtained as follows:

(13) $$\{\begin{array}{c} \left\|P_{A}-P_{C}\|=\|P_{B}-P_{C}\right\|\\ \frac{x_{A}}{z_{A}}=\frac{x_{B}}{z_{B}}\\ \frac{y_{A}}{z_{A}}=\frac{y_{B}}{z_{B}} \end{array}$$

where $P_{A}\left(x_{A},y_{A},z_{A}\right)$ is the 3D coordinates of A, $P_{C}$ is the 3D coordinates of C, and $P_{B}\left(x_{B},y_{B},z_{B}\right)$ is the 3D coordinates of B. The first term represents the 3D distance constraint and the second and third term represent the projection constraint. The unique solution $P_{B}$ can be obtained by solving the above equations.

#### 3.3.2. Upper Body Reverse Joint Correction Algorithm

(1) Correction algorithm of Neck point based on increasing confidence

**Figure 5:**
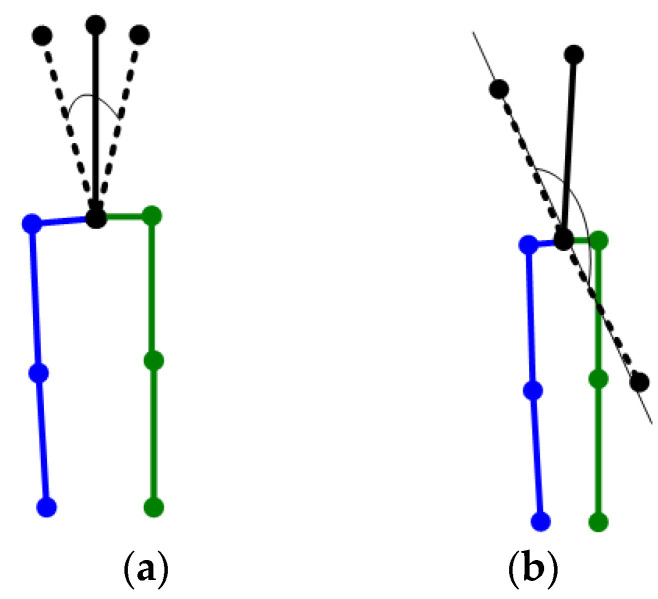
The reasonable range of the spine. (**a**) Left and right range. (**b**) Front and back range. On every subgraph, the reasonable range is between two black dashed lines.

The depth ambiguity is also reflected in the Neck point. The depth of Neck can be either larger or smaller than the depth of Waist. The rules for Neck correction are as follows: (1) The angle between the spine and the two hips can not be too small; (2) the angle between the spine and legs should be limited, as shown in Figure 5.

In order to enhance the robustness of the algorithm, we design a confidence function combining the above rules. An increasing confidence strategy is used, that is, when the confidence of the corrected pose is greater than that of the last one, this correction will be adopted. The confidence function is as follows:

(14) $$b=b_{\alpha }+b_{\beta }$$

(15) $$b_{\alpha }=\frac{1}{e^{1-t_{1}}-1}\left(1-e^{cos\alpha -t_{1}}\right)$$

**Figure 6:**
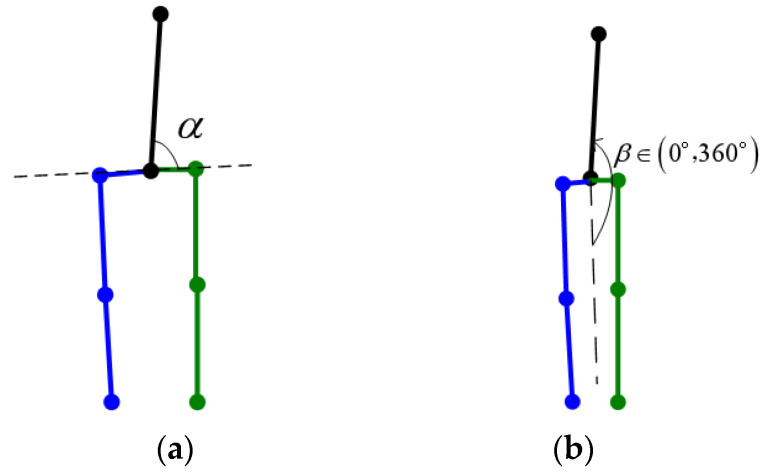
The angles of the spine. (**a**) α for rule (1); (**b**) β for rule (2).

$b_{\alpha }$ corresponds to the rule (1). α is the angle between the spine and the line connecting both hips, as shown in Figure 6a. $t_{1\;}\;$ is a threshold set to cos80°. The confidence is positive in the interval [80°, 90°]. When the angle is less than 80°, the confidence will become negative and decline rapidly.

$b_{\beta }\;$ is the confidence function for rule (2), where β∈(0°,360°)  is shown in Figure 6b, when the angle between the spine and the dashed line is less than 30°, or more than 210°, the confidence will decline rapidly.

(16) $$b_{\beta }=\{\begin{array}{ll} -\frac{cos\beta }{1-cost_{2}}+\frac{1}{1-cost_{2}}-1, & \beta <30{}^{\circ}\\ \frac{\cos\left(\beta -90{}^{\circ}\right)}{1+cost_{3}}+\frac{1}{1+cost_{3}}-1, & \beta >210{}^{\circ}\\ \;\;0.15, & else \end{array}$$

**Figure 7:**
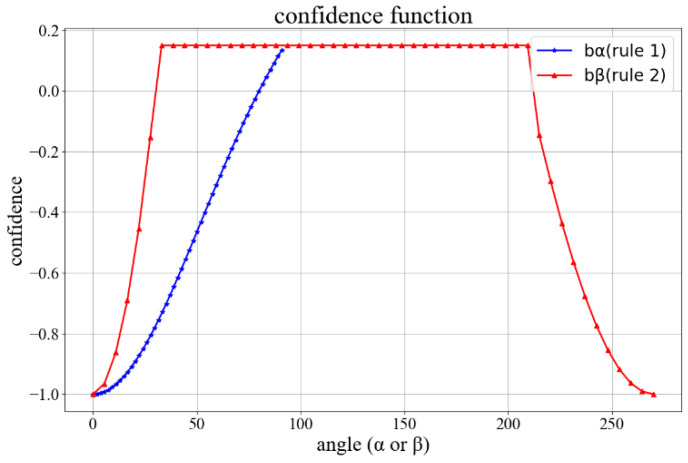
The curve of two confidence functions.

The curves of $b_{\alpha }$ and $b_{\beta }$ are shown in Figure 7.

(2) Correction algorithm for reverse joints of arms

In all kinds of human postures, the movement of arms is the most complex. Similar to legs, the corresponding detection and correction rules are set for arms.

**Figure 8:**
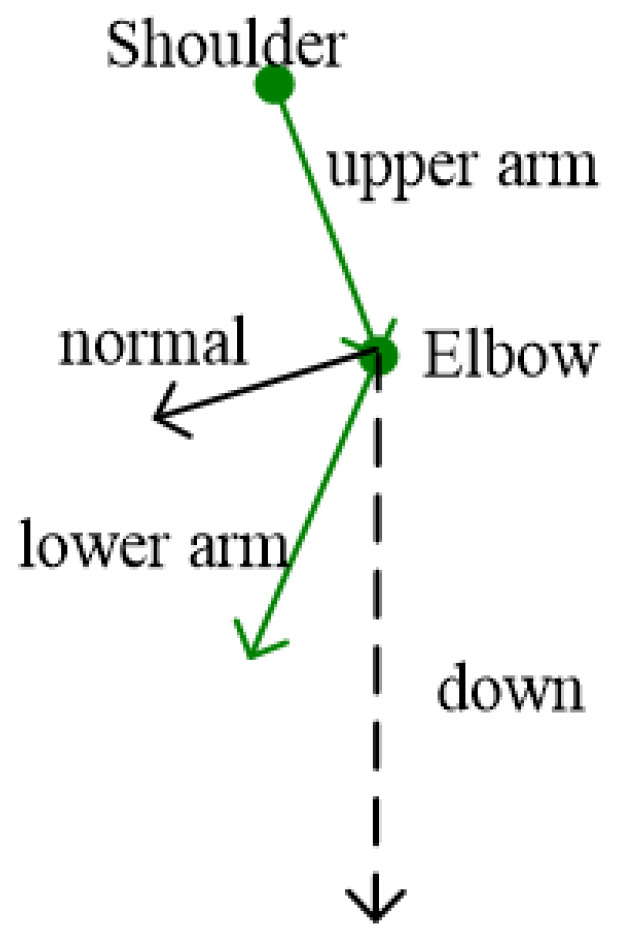
Vectors and points in correction algorithm for arms.

The normal direction of the arms is taken as the constraint condition. The “normal direction” is defined as the upper arm vector crossing the lower arm vector, as shown in Figure 8.

The angle between the normal of the left arm and the “down” direction is no more than 105°, and the angle between the normal of the right arm and the “up” direction is no more than 105°. The specific implementation is similar to legs correction. In the possible pose space, appropriate poses are searched successively, as shown in Figure 4, and described in Section 3.3.1.

#### 3.3.3. Reverse Joint Suppression Algorithm

(1) For legs

**Figure 9:**
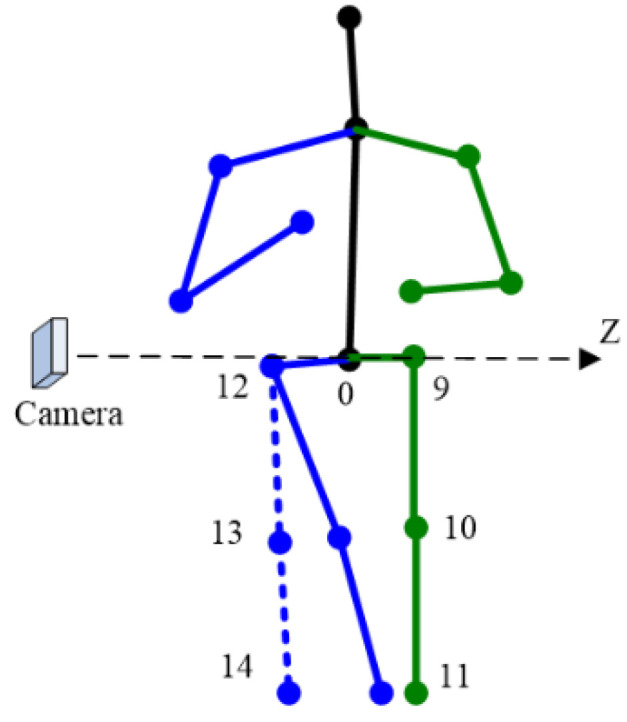
Drifted right leg under side view.

In addition to the above correction algorithms, we studied a reverse joint suppression algorithm to avoid the legs “drift” under the side view. As is shown in Figure 9, the dashed line of the right leg represents the correct pose, while the solid line is the “drifted” pose.

In the standing posture, the angle between the thigh and the line connecting two hips tends to be 90°. Thus, in our algorithm, when the angle is less than 80°, the penalty term will be added.

Take the right knee as an example, the loss function:

(17) $$E_{Rleg}=S_{1}\left(f\left(P_{13}\right)\right)$$

where $P_{13}$ denotes the 3D coordinates of Right Knee, and *f*( ) is used as a measure of angle described above:

(18) $$f\left(P_{13}\right)={\left(\frac{P_{13}-P_{12}}{\left\|P_{13}-P_{12}\right\|}\cdot \frac{P_{12}-P_{9}}{\left\|P_{12}-P_{9}\right\|}\right)}^{2}$$

**Figure 10:**
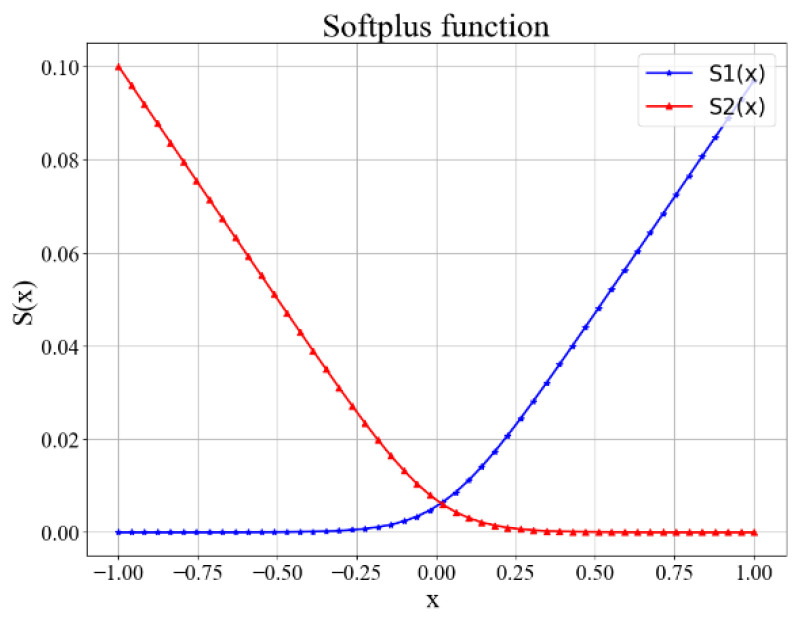
Curves of two Softplus functions.

$P_{12},\;P_{9}$ denote 3D coordinates of two Hips (known). $S_{1}\left(x\right)$ is the Softplus function:

(19) $$S_{1}\left(x\right)=\frac{1}{\gamma }\ln\left(1+e^{\beta \left(x-t_{4}\right)}\right)$$

where γ,  β, *t* are super parameters. When $\gamma =100,\beta =10,\;t_{4}=cos^{2}80{}^{\circ}$. The curve of the $S_{1}\left(x\right)$ function is shown in Figure 10. Furthermore, there is another kind of Softplus function used in our algorithm–$S_{2}\left(x\right)$, when we expect x to be no less than some threshold.

When the value of x is less than $t_{4}$, the curve of $S_{1}\left(x\right)$ is gentle, and when *x* exceeds it, the value of the function will increase rapidly.

(2) For arms

**Figure 11:**
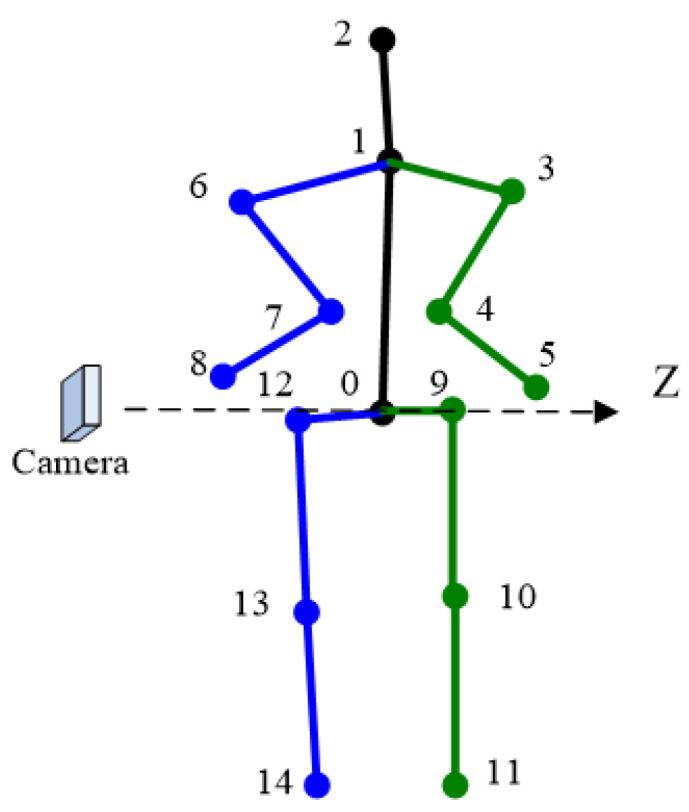
Reverse joints of arms to be suppressed.

Mostly, the elbows are on the outside of shoulders. When elbows are on the inside of shoulders (as shown in Figure 11), the penalty term will be added. The loss function is:

(20) $$E_{arm}=S_{2}\left(x\right)=\frac{1}{\gamma }\ln\left(1+e^{-\beta x}\right)$$

For the left arm $x=r(P_{4z}-P_{3z})$, for the right $x=r(P_{6z}-P_{7z})$, r is the 3D human orientation estimated by Equation (11). When r > 0, the depth of Left Shoulder is larger than that of Right Shoulder, we expect the depth of Left Elbow is larger than that of Left Shoulder, and depth of Right Elbow is smaller than that of Right Shoulder. When r < 0, the case is just the opposite. Anyway, we want x > 0, so $S_{2}\left(x\right)$ is used as a loss function. The curve of $S_{2}\left(x\right)\;$ is shown in Figure 9. γ,β are the same as those in $S_{1}\left(x\right)$.

### 3.4. Graph Optimization Algorithm Based on Multiple Constraints

Many problems in robotics and computer vision can be solved by least-squares optimization methods, e.g., SLAM [32] or bundling adjustment (BA) [33]. Graph optimization refers to the representation of optimization problems in the form of graphs. In this paper, we utilize the generic graph optimization (G2O [34]) framework, which is widely used in SLAM. Each vertex in the graph represents a variable to be optimized, and each edge on the graph represents a constraint (loss function) on the vertices it connects. The optimization problem can be solved by Levenberg–Marquardt (LM) algorithm [35,36], Dogleg method [37], etc.

**Figure 12:**
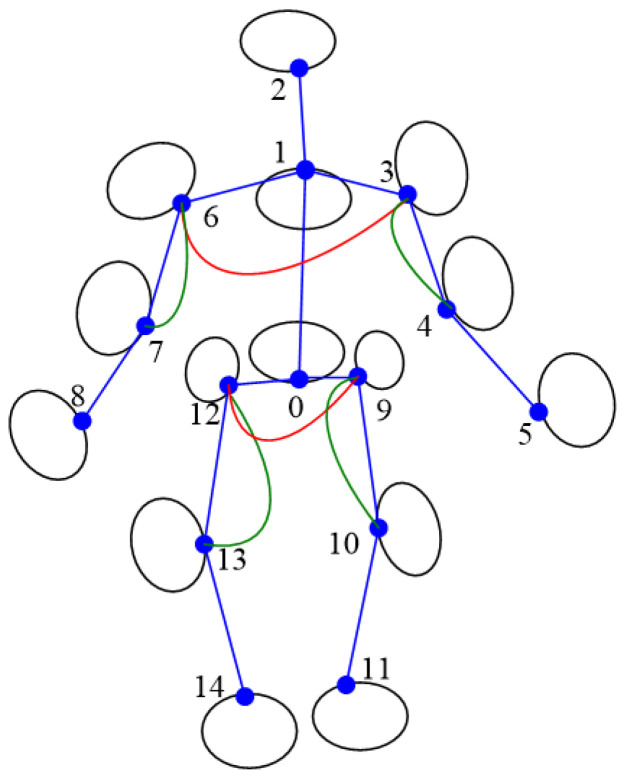
Graph model in our algorithm. Black edges denote the projection constraints, blue ones denote the bone length constraints, red ones denote the 3D orientation constraints and green ones denote the reverse joint constraints. The inverse joint correction algorithm is not shown in the figure because it is not an optimization algorithm.

The graph constructed in this paper is as shown in Figure 12. The vertices represent the 3D keypoints to be solved. The edges in the graph model correspond to constraints. There are edges that connect two vertices, called binary edges. Edges (black) connected to only one vertex are called unary edges. Different edges are represented by different colors.

The loss function of the graph optimization least-squares problem is:

(21) $$E_{G}=E_{R}+E_{L}+E_{O}+E_{leg}+E_{arm}$$

where $E_{R}\;$ represents the reprojection loss, $E_{L}$ the bone length loss, $E_{O}$ the 3D human body orientation loss and $E_{leg},\;E_{arm}$ the reverse joint suppression loss of legs and arms, respectively.

(1) Reprojection loss
  (22) $$E_{R}=\frac{1}{2}\underset{i}{\sum }f_{i}^{R}{\left(P_{i}\right)}^{2}=\frac{1}{2}\underset{i}{\sum }{\left({\hat{R}}_{i}-R_{i}\right)}^{2}$$
  where $R_{i}\;$ is the coordinate of the ith 3D keypoint projected onto the 2D image according to the pinhole camera model; ${\hat{R}}_{i}\;$ is the 2D keypoint extracted through CPN [9] or other methods; $P_{i}\;\left(x_{i},y_{i},z_{i}\right)$ is the coordinate of the 3D keypoint to be solved; K is the intrinsic matrix of the camera; $f_{i}^{R}\left(P_{i}\right)$ is the projection error of the keypoint.
(2) Length loss
  (23) $$E_{L}=\frac{1}{2}\sum_{j}{\left({\hat{L}}_{j}-L_{j}\right)}^{2}=\frac{1}{2}\sum_{j}{\left({\hat{L}}_{j}-\|P_{k}-P_{l}\|\right)}^{2}.$$
  where ${\hat{L}}_{j}$ represents the 3D bone length solved according to Section 3.1.2, and $L_{j}$ is the current distance between keypoints the bone connected ($P_{k}\;\mathrm{and}\;P_{l})$.
(3) Orientation loss
  (24) $$E_{O}=E_{O1}+E_{O2}+E_{A}$$
  where $E_{O1}$ denotes the upper body orientation constraint and $E_{O2}$ denotes the lower body constraint.
  (25) $$E_{O1}=S_{2}\left(r\cdot \left(P_{3z}-P_{6z}\right)\right)$$
  (26) $$E_{O2}=S_{2}\left(r\cdot \left(P_{9z}-P_{12z}\right)\right)$$
  where $S_{2}\left(x\right)$ is defined in Equation (20), r is defined in Equation (11), $P_{3z}$, $P_{6z}$, $P_{9z}$, $P_{12z}$ denotes the depth of Left Shoulder, Right Shoulder, Left Hip and Right Hip, respectively. When r > 0, the depths of left limbs are expected to be larger than those of right limbs and vice versa.

$E_{A}$ is the shoulder–hip angle constraint. Mostly, the line connecting two Shoulders and the one connecting two Hips tend to be parallel.

(27) $$E_{A}=S_{2}\left(\frac{P_{3}-P_{6}}{\left\|P_{3}-P_{6}\right\|}\cdot \frac{P_{9}-P_{12}}{\left\|P_{9}-P_{12}\right\|}-t_{5}\right)$$

where $t_{5}$ is a threshold set as $t_{5}=0.9$. $P_{3}$, $P_{6}$, $P_{9}$, $P_{12}$ denote 3D coordinates of Left Shoulder, Right Shoulder, Left Hip and Right Hip, respectively.

To maintain the robustness of the algorithm, only the 3D coordinates of the two Shoulders are optimized here.

(4) Reverse joint loss

$E_{leg},E_{arm}$ are defined in Section 3.3.3.

LM algorithm [35,36] was used to optimize the graph model. In addition, the reverse joint correction loss is expressed as:

(28) $$E^{R}=E_{neck}^{R}+E_{arm}^{R}+E_{leg}^{R}$$

where $E_{neck}^{R},E_{arm}^{R},E_{leg}^{R}$ denote the correction losses of neck, arms and legs, respectively. The total losses of the overall algorithm are:

(29) $$E=E^{R}+E_{G}$$

## 4. Results

In this section, we validate our G2O-pose method with the Human3.6m [22] dataset. Section 4.1, Section 4.2 and Section 4.3 are separate evaluation results of Section 3.1, Section 3.2, Section 3.3 and Section 4.4 is the evaluation of the total algorithm with the metric MPJPE. All experiments are performed on a consumer laptop with an Intel i7 1.8G CPU, 8 GB RAM and a GeForce MX250 GPU.

### 4.1. Bone Proportion and Length Recovery

#### 4.1.1. Bone Segment Proportions

For Human3.6m, the commonly used subjects are $S_{1},S_{5},S_{6},S_{7},S_{8},S_{9},S_{11}$. We select actions with more upright postures for estimation, such as *Directions*, *Greeting*, *Waiting*, *Posing*, *Waiting*, *WalkTogether*, etc. The metric is defined as the average absolute distance between the calculated proportions and the ground truth:

(30) $$d\left(s,s^{gt}\right)=\frac{1}{14}\underset{i}{\sum }\left|s_{i}-s_{i}^{gt}\right|,i=0,1,\ldots 13$$

where $s^{gt}$ denotes the ground truth, | | denotes the absolute value. Both the 2D keypoints extracted by CPN [9] and ground truth 2D keypoints are used for the experiment. Results are shown in Table 2 and Table 3.

#### 4.1.2. Bone Segment Lengths

In the initial frame of the dataset, the body is relatively stretched. Therefore, only the first 10 frames in each view are used to calculate the bone length. To simplify the calculation, only four actions (Directions 1, Directions, Discussion 1, Discussion) are used.

The average bone length error is:

(31) $$e=\frac{1}{14}\underset{i}{\sum }\left|\frac{d^{gt}}{d^{s}}L_{i}-L_{i}^{gt}\right|,i=0,1,\ldots 13$$

where $L_{i}^{gt}$ denotes the ground truth, $L_{i}$ denotes the calculated length by 3.1.2, $d^{s}$ denotes the supposed depth of the Waist point as described in 3.1.2 and $d^{gt}$ denotes the ground truth of the Waist point. Results are shown in Table 4.

### 4.2. Classification of Body Orientation

The ground truth orientation of the body is calculated by:

(32) $$F^{gt}=down\times left$$

where *down* is defined by unit vector (0,1,0) in the camera coordinate system; *left* represents the left direction to the human body itself. The calculation method is as follows:

(33) $$left=P_{3}-P_{6}+P_{9}-P_{12}$$

where $P_{3},P_{6},P_{9},P_{12}$ denote the ground truth 3D coordinates of the Left Shoulder, Right Shoulder, Left Hip and Right Hip, respectively, in the camera coordinate system.

The metric is defined as:

(34) $$p=\frac{1}{N}\sum_{i}(F_{i\left(x\right)}^{gt}\cdot r_{i})>0$$

where *N* denotes the total number of the frames. Subscript (*x*) denotes the *x* coordinate of vector $F_{i}^{gt}$. *r* is calculated by Equation (11), and the weight is defined as:

(35) $$w_{1}=w_{4}=w_{5}=1,w_{2}=w_{3}=0.5$$

**Figure 13:**
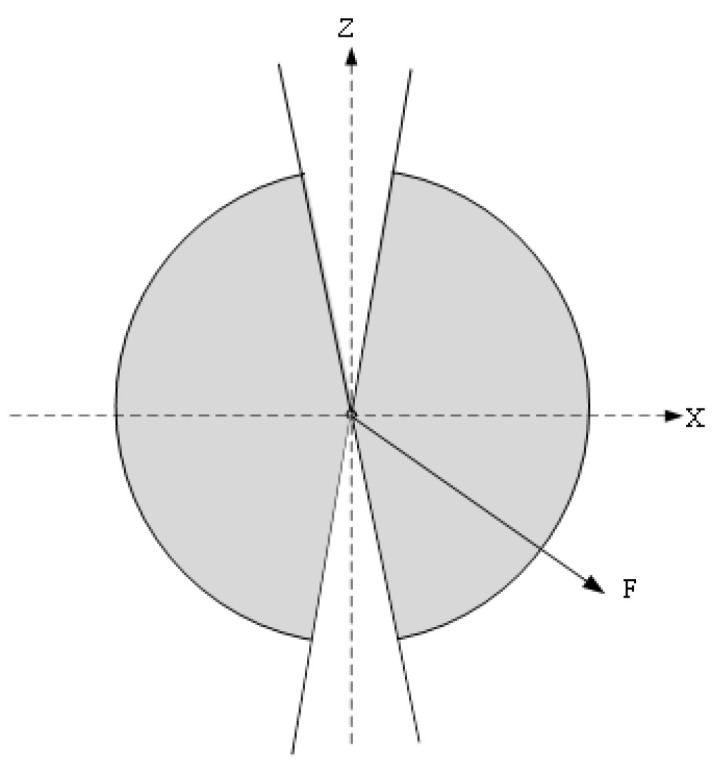
The result will be counted when F point to the shaded area.

When $r_{i}$ has the same plus–minus sign with $F_{i\left(x\right)}^{gt}$, the orientation predicted is considered correct. In addition, the orientation around the direction consistent with the Z-axis has some ambiguity, and this does not have much influence on the 3D pose. Only when the angle between it and the Z-axis is more than 5° (as shown in Figure 13), *p* is calculated.

**Figure 14:**
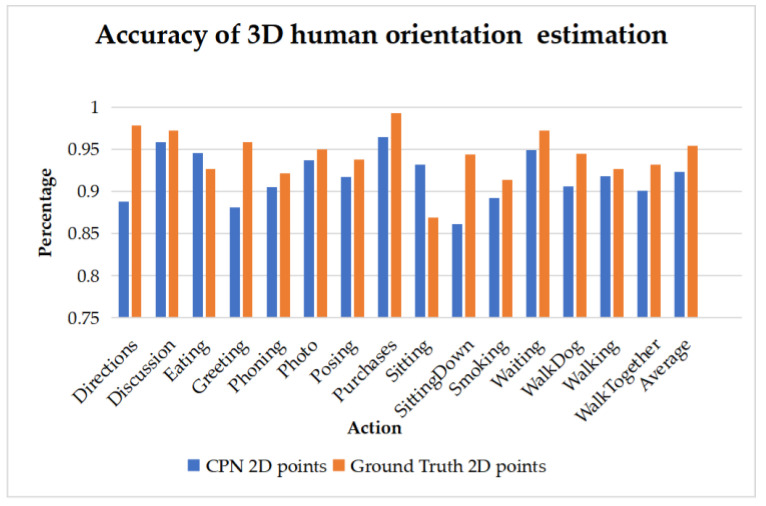
The result will be counted when F points to the shaded area.

Statistics result according to actions is shown in Figure 14. The horizontal axis is for 15 different actions, the vertical axis is as the estimation accuracy. Both CPN 2D keypoints and ground truth 2d keypoints are tested. The average accuracy is 92.3% and 95.4%, respectively.

### 4.3. Reverse Joint Correction

The correction success rate is used to test the effect of the algorithm. When the corrected pose is closer to the ground truth, the correction is considered successful. The distance is defined as:

(36) $$d=\underset{i}{\sum }\|\frac{d^{gt}}{d^{s}}(P_{i}-P_{0})-(P_{i}^{gt}-P_{0}^{gt})\|,i=0,1,\ldots 14$$

where $P_{i}\;$ denotes the coordinate of corrected 3D keypoints; $P_{i}^{gt}$ is the corresponding ground truth. $d^{s}$ denotes the supposed initial depth of the Waist point; $d^{gt}\;$ denotes the corresponding ground truth. Success rates of corrections are shown in Table 5.

### 4.4. Three-Dimensional Human Pose Estimation Results

In the Human3.6m dataset, the subjects $S_{1},S_{5},S_{6},S_{7}$,$S_{8}$ are usually used for training, and $S_{9}$, $S_{11}$ are usually used for testing. We choose a portion of the dataset for verification. Only the frames with little depth change (<0.3 m) relative to the initial are counted. The initial depth of Waist point is supposed as 5 m for all actions of the two subjects. We compare our algorithm with [5,19,20,21], with the given 2D points of the dataset. The results are shown in Table 6; the metric is MPJPE, defined as the average error of per joint after alignment of the Waist point and scaling to the same scale as the ground truth:

(37) $$e=\frac{1}{14}\underset{i}{\sum }\|\frac{d^{gt}}{d^{s}}(P_{i}-P_{0})-\left(P_{i}^{gt}-P_{0}^{gt})\right\|,i=1,\ldots 14$$

where $P_{i}\;$ denotes the coordinate of the 3D keypoints; $P_{i}^{gt}$ is the corresponding ground truth. $d^{s}$ denotes the supposed initial depth of the Waist point; $d^{gt}\;$ denotes the corresponding ground truth. The “average” in our algorithm is a weighted average according to the number of frames of each action, due to the frame number varying greatly.

Although the MPJPE of our algorithm is a little higher than that of Monocap, the running speed of ours is much faster. The comparison of running speeds is shown in Table 7. The average FPS of the proposed algorithm reaches 32. In addition, because the algorithm adopts interval sampling, which is calculated every 4 frames, the frame rate of the original 2D keypoints can reach more than 100 fps. The running speed of the proposed method mainly benefits from utilizing the previous 3D pose for initializing the next frame, rather than treating them separately. Thus, only a minor adjustment to the initial pose is required due to slight movements between frames.

**Figure 15:**
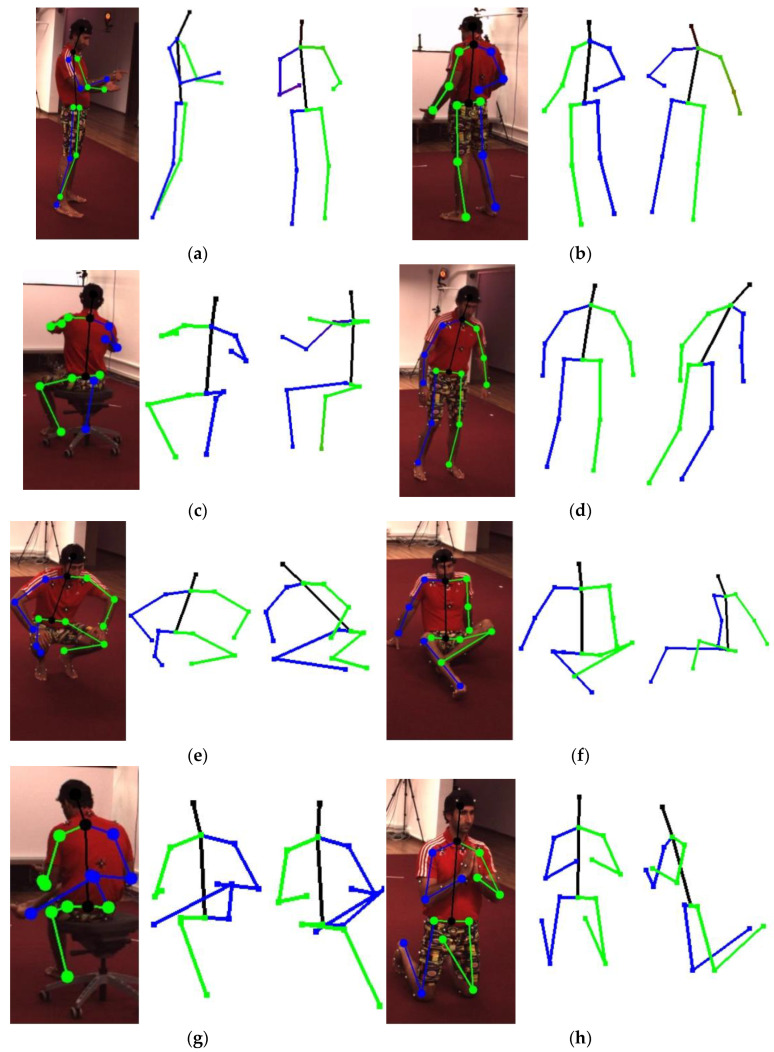

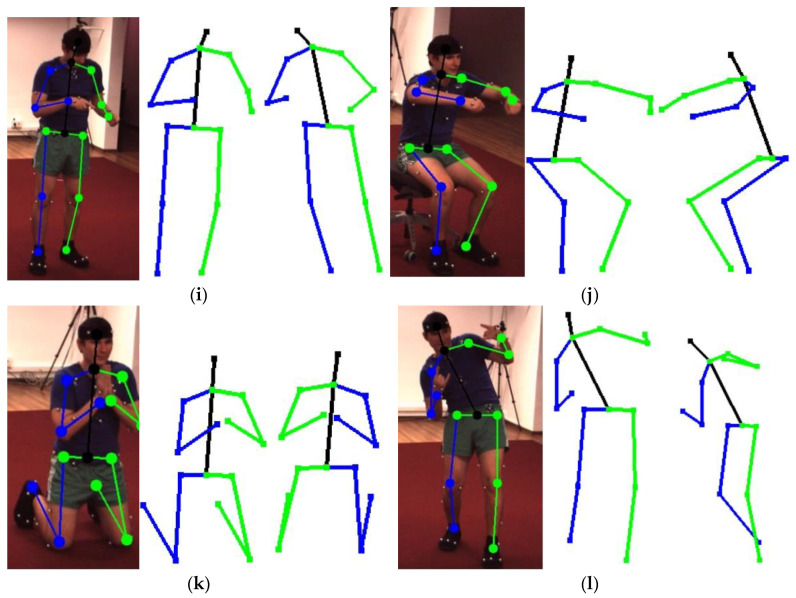
The visualization of G2O-pose. Subfigures (**a**–**l**) are the experimental results selected from the various actions of the two subjects. In every subfigure, a 2D image and 2D pose, 3D pose, and 3D pose from another random perspective are shown in sequence.

The visualization results are shown in Figure 15, which are presented using the OpenGL [38] open source library.

## 5. Discussions

### 5.1. Analysis about the Loss Functions

**Figure 16:**
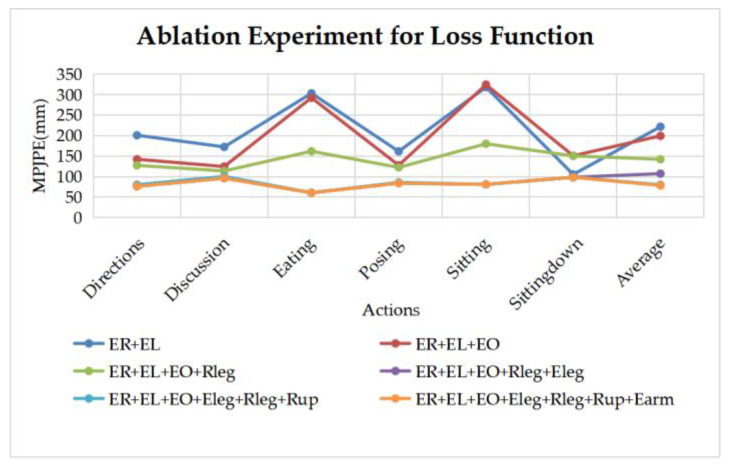
The ablation results of different losses. E_R_, E_L_, E_O_, E_leg_, E_arm_ are described in Equation (21), Section 3.4; R_leg_, R_up_ denote the reverse joint correction loss of legs and upper body described in Section 3.3.

In this subsection, we will perform ablation experiments to compare the effect of each loss function on MPJPE. As shown in Figure 16, most errors decrease gradually with the addition of the loss terms, although different curves occasionally cross over. The reverse joint correction algorithms have obvious effects on the complex actions, e.g., *Eating*, *Sitting*. The last two curves are too close to each other to see clearly; their average MPJPE differs by 2 mm.

### 5.2. Sensitivity to the Depth Range of the Subject

**Figure 17:**
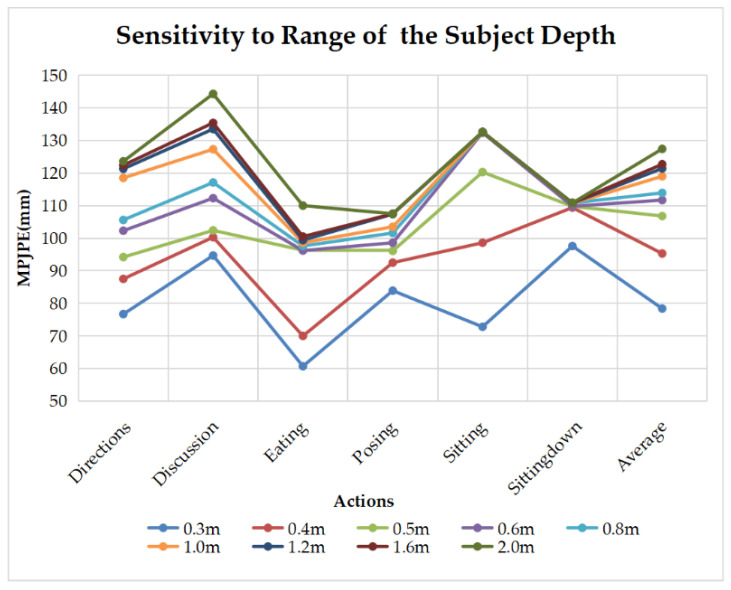
Sensitivity to range of the subject depth.

We found that when the depth of the body is out of the range, the errors are mainly related to the angles of bone rotation. Specifically, our algorithm infers the 3D pose through the changes of 2D keypoints. Both the bone rotation and the depth change of the subject will lead to changes of 2D keypoints. Take the arm, for example: when it is lifted from the vertical position, or when the subject is moving further from the camera, the length of the arm on the image will get shorter. If the depth change of the subject can not be accurately inferred, the G2O optimizer of our algorithm will be confused between the “depth change” and “rotation change”. The detailed errors induced from depth range are shown in Figure 17. The average MPJPE ranges from 78.4 mm to 127.4 mm as the depth range goes from 0.3 m to 2 m.

### 5.3. About the Initial Supposed Depth

**Figure 18:**
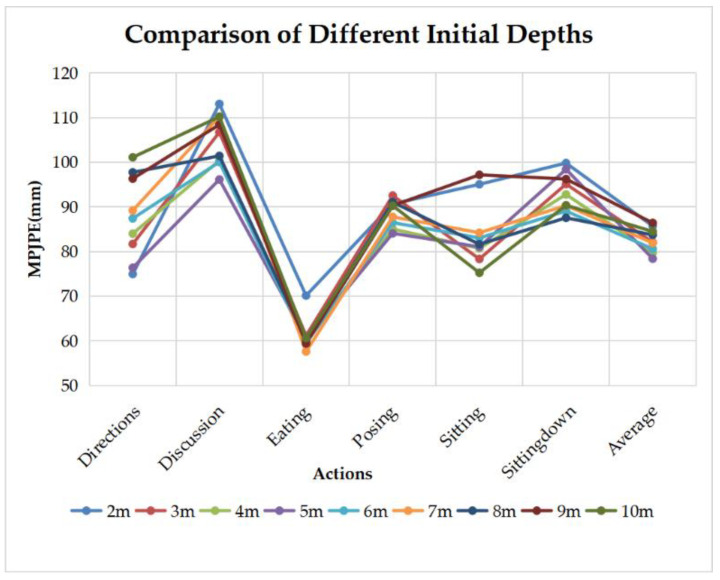
Comparison of different supposed initial depths.

In our experiment, we suppose the initial depth of Waist point of the subject as 5 m, while the ground truth ranges from 3.92–5.68 m. For more general cases, we test the choice of initial depth from 2–10 m on the same datasets, the results do not show much difference in average MPJPE, as shown in Figure 18.

Although the algorithm in this paper has achieved certain effects, there are still some limitations and improvements. The algorithm is only applicable to the scene where the depth of the human body does not change much, the recovery of 3D bone proportions needs to select poses with more upright actions, and the reverse joint correction algorithm is only applicable to daily human activities and is not applicable to difficult and large movements. Despite the shortcomings, our algorithm has a high speed for real-time application.

## 6. Conclusions

Monocular 3D human pose estimation has the problem of ambiguity along the depth direction. In this paper, we propose a G2O-pose based on graph optimization to solve the problem of low efficiency of traditional methods. Our method achieves real-time performance through the following algorithms: (1) a 3D bone proportion recovery algorithm based on 2D keypoints; (2) a 3D human orientation classification algorithm based on weighted 2D joint features; (3) a reverse joint correction algorithm based on a heuristic search; (4) a reverse joint suppression algorithm based on human joint rotation angles. The accuracy is slightly lower than the previous traditional method, while the speed is much faster. In future works, we will study the method of solving the depth change to make the algorithm more applicable.

## Figures and Tables

**Table 1:** 2D vectors description.

Symbol	Start	End	Symbol	Start	End
$L_{1}$	neck	head	$L_{13}$	right knee	right ankle
$L_{6}$	right shoulder	right elbow	$L_{9}$	left hip	left knee
$L_{7}$	right elbow	right wrist	$L_{10}$	left knee	left ankle
$L_{3}$	left shoulder	left elbow	$L_{12}$	right hip	right knee
$L_{4}$	left elbow	left wrist			

**Table 2:** Results of Bone proportion recovery (CPN 2D points). The spine is set as scale 1. GPR is the method proposed by [31] (Bold indicates higher accuracy).

Subject	$S_{1}$	$S_{5}$	$S_{6}$	$S_{7}$	$S_{8}$	$S_{9}$	$S_{11}$	Average
GPR	0.072	0.078	0.089	0.083	0.052	0.073	0.055	0.072
ours	**0.027**	**0.038**	**0.035**	**0.038**	**0.018**	**0.023**	**0.045**	**0.032**

**Table 3:** Results of Bone proportion recovery (ground truth 2D points). The spine is set as scale 1. GPR is the method proposed by [31] (Bold indicates higher accuracy).

Subject	$S_{1}$	$S_{5}$	$S_{6}$	$S_{7}$	$S_{8}$	$S_{9}$	$S_{11}$	Average
GPR	0.055	0.048	0.057	0.049	0.032	0.041	0.035	0.045
ours	**0.013**	**0.011**	**0.009**	**0.017**	**0.010**	**0.008**	**0.012**	**0.012**

**Table 4:** Results of bone length recovery (mm).

Subject	$S_{1}$	$S_{5}$	$S_{6}$	$S_{7}$	$S_{8}$	$S_{9}$	$S_{11}$	Average
CPN 2D	10.1	6.0	6.7	7.3	7.1	10.2	12.3	8.5
GT 2D	4.8	2.6	3.8	4.5	3.7	5.1	5.7	4.3

**Table 5:** Results of reverse joint correction experiment.

Left Leg	Right Leg	Neck	Left Arm	Right Arm	Average
84.4%	94.6%	75.4%	84.6%	80.0%	83.8%

**Table 6:** MPJPE of the algorithms given 2D keypoints (mm) (Bold indicates highest accuracy).

Action	Directions	Discussion	Eating	Posing	Sitting
PMP [19]	126.7	133.5	124.7	136.2	146.1
NRSFM [20]	136.5	139.6	134.3	157.2	181.7
Convex [21]	94.3	89.2	85.4	96.1	80.1
Monocap [5]	**73.1**	**69.2**	82.0	**73.7**	**71.4**
Ours	76.7	94.7	**60.7**	83.9	72.8
Action	Sitting Down	Average			
PMP [19]	162.4	134.4			
NRSFM [20]	176.9	150.7			
Convex [21]	97.4	90.4			
Monocap [5]	**90.3**	**76.4**			
Ours	97.6	78.4			

**Table 7:** Running speed of various algorithms (FPS) (Bold indicates fastest).

Action	Directions	Discussion	Eating	Posing	Sitting	Sitting Down	Total
PMP [19]	0.22	0.21	0.21	0.22	0.22	0.23	0.22
NRSFM [20]	<0.1	<0.1	<0.1	<0.1	<0.1	<0.1	<0.1
Convex [21]	0.52	0.51	0.51	0.52	0.50	0.51	0.51
Monocap [5]	0.85	0.84	0.86	0.84	0.85	0.86	0.85
Ours	**31.43**	**32.51**	**33.60**	**32.38**	**32.81**	**31.77**	**32.54**

## Data Availability

Publicly available datasets were analyzed in this study. These data can be found here: http://vision.imar.ro/human3.6m/description.php (accessed on 15 February 2022).
